# Conditional deletion of ROCK2 induces anxiety-like behaviors and alters dendritic spine density and morphology on CA1 pyramidal neurons

**DOI:** 10.1186/s13041-021-00878-4

**Published:** 2021-11-18

**Authors:** Audrey J. Weber, Ashley B. Adamson, Kelsey M. Greathouse, Julia P. Andrade, Cameron D. Freeman, Jung Vin Seo, Rosaria J. Rae, Courtney K. Walker, Jeremy H. Herskowitz

**Affiliations:** Center for Neurodegeneration and Experimental Therapeutics, Department of Neurology, University of Alabama at Birmingham, 1825 University Blvd, Birmingham, AL 35294 USA

**Keywords:** ROCK2, Rho kinase, Dendritic spine, Hippocampus, Prefrontal cortex, Amygdala

## Abstract

Rho-associated kinase isoform 2 (ROCK2) is an attractive drug target for several neurologic disorders. A critical barrier to ROCK2-based research and therapeutics is the lack of a mouse model that enables investigation of ROCK2 with spatial and temporal control of gene expression. To overcome this, we generated ROCK2^fl/fl^ mice. Mice expressing Cre recombinase in forebrain excitatory neurons (*CaMKII*-Cre) were crossed with ROCK2^fl/fl^ mice (Cre/ROCK2^fl/fl^), and the contribution of ROCK2 in behavior as well as dendritic spine morphology in the hippocampus, medial prefrontal cortex (mPFC), and basolateral amygdala (BLA) was examined. Cre/ROCK2^fl/fl^ mice spent reduced time in the open arms of the elevated plus maze and increased time in the dark of the light–dark box test compared to littermate controls. These results indicated that Cre/ROCK2^fl/fl^ mice exhibited anxiety-like behaviors. To examine dendritic spine morphology, individual pyramidal neurons in CA1 hippocampus, mPFC, and the BLA were targeted for iontophoretic microinjection of fluorescent dye, followed by high-resolution confocal microscopy and neuronal 3D reconstructions for morphometry analysis. In dorsal CA1, Cre/ROCK2^fl/fl^ mice displayed significantly increased thin spine density on basal dendrites and reduced mean spine head volume across all spine types on apical dendrites. In ventral CA1, Cre/ROCK2^fl/fl^ mice exhibited significantly increased spine length on apical dendrites. Spine density and morphology were comparable in the mPFC and BLA between both genotypes. These findings suggest that neuronal ROCK2 mediates spine density and morphology in a compartmentalized manner among CA1 pyramidal cells, and that in the absence of ROCK2 these mechanisms may contribute to anxiety-like behaviors.

**Supplementary Information:**

The online version contains supplementary material available at 10.1186/s13041-021-00878-4.

## Introduction

Rho-associated coiled-coil containing kinase isoform 2 (ROCK2) belongs to the AGC serine/threonine kinase family and was originally identified as an interacting partner of GTP-bound RhoA. Two mammalian ROCK isoforms exist, ROCK1 and ROCK2, and they share 92% amino acid identity in their kinase domains but only 65% amino acid similarity in the carboxyl-terminus regions [1]. ROCK1 and ROCK2 are established mediators of actin–myosin cytoskeleton contractility [1–5]. While both kinase isoforms share similar expression patterns, ROCK1 transcript levels are most abundant in thymus and blood, and ROCK2 is most prominent in brain [6]. ROCK1 and ROCK2 phosphorylate a number of substrates that are involved in the regulation of cellular morphology and motility, as well as cell–cell adhesion. Currently, ROCKs are promising drug targets for several human disorders, including insulin resistance, asthma, kidney failure, erectile dysfunction, osteoporosis, and cancer [7–12]. Notably, special emphasis is placed on ROCK2 as a target for neurologic conditions, including Alzheimer’s disease, Frontotemporal Dementia, Parkinson’s disease, Amyotrophic Lateral Sclerosis, glaucoma, and stroke [13–22].

The majority of research to understand the role of ROCKs in brain has utilized pan-ROCK small-molecule inhibitors, with Y27632 and Fasudil being most commonly employed. These drugs, as well as other current generation ROCK inhibitors, are not isoform-selective and likely inhibit other AGC kinases, especially at doses used for in vivo studies [23]. Genetic approaches, such as RNAi, in cell-based assays suggest distinct functions of ROCK isoforms [24–27]. However, dissecting the role of ROCK isoforms in vivo has been challenging. Conventional homozygous knockout mice exhibit severe developmental abnormalities or embryonic lethality in ROCK1^−/−^ or ROCK2^−/−^ mice, respectively [28–30]. Studies using heterozygous models revealed that ROCK1^+/−^ and ROCK2^+/−^ mice develop normally and general health, weight, in-cage behavior, and food consumption were similar among heterozygous mice and their littermate controls [21, 30–32]. Previous work indicated that adult male and female ROCK2^+/−^ mice displayed anxiety-like behaviors on the elevated plus maze (EPM) compared to ROCK2^+/+^ littermates, which was similar to wild-type mice that were treated with the pan-ROCK inhibitor Fasudil for 30 days [32]. While rodent avoidance of the open arms in the EPM is associated with alterations in the firing rate of single neurons in the medial prefrontal cortex (mPFC) [33], ROCK2^+/−^ mice exhibited only modest changes in dendritic spine morphology among layer 2–3 pyramidal neurons of the mPFC [31]. This suggests that the neurobiological mechanisms driving the anxiety-like behaviors in ROCK2^+/−^ mice remain to be fully elucidated. For instance, it is possible that (1) different and/or additional brain regions contribute to the anxiety-like behaviors or that (2) peripheral, non-central nervous system effects of ROCK2 heterozygosity are responsible. Alternatively, ROCK2 reduction in non-neuronal cells of the brain may have impacted the phenotypes that were observed. These are critical questions to address given that Fasudil and other ROCK inhibitors, including the more selective ROCK2 inhibitor KD025, are being used in ongoing clinical trials (see https://www.clinicaltrials.gov).

Despite the aforementioned caveats of the ROCK2^+/−^ mice, mounting evidence suggests that ROCK2 likely influences cognitive behavior through regulation of the actin cytoskeleton and ultimately structural plasticity of dendritic spines. Studies in primary cultured hippocampal neurons indicated that over-expression of ROCK2 significantly reduced dendritic spine density, while RNAi-mediated reduction of ROCK2 marginally increased spine density [27]. Validating these findings in vivo has been challenging due to the lack of a mouse model that enables investigation of ROCK2 with cell-type control of gene expression. To overcome this, we generated ROCK2^fl/fl^ mice and examined how deletion of ROCK2 in excitatory neurons impacted cognitive behavior as well as dendritic spine density and morphology in three different brain regions. The findings suggest a novel role for ROCK2 in CA1 pyramidal neurons of the hippocampus and provide behavioral implications for these mechanisms.

## Methods

### Animals

All experimental procedures were performed under a protocol approved by the Institutional Animal Care and Use Committee at the University of Alabama at Birmingham. Mice were housed 5–7 per cage on a 12 h light/dark cycle and had ad libitum access to food and water. Both male and female mice were used. ROCK2^fl/fl^ mice were generated as follows: C57BL/6N-Rock2tm1a(KOMP)Wtsi mice were made from ES cells purchased from the International Mouse Phenotyping Consortium at the University of California, Davis. ES cell injections were performed by the UAB Transgenic & Genetically Engineered Models Core. The C57BL/6N-Rock2tm1a(KOMP)Wtsi ES cells used for this research project were generated by the trans-NIH Knock-Out Mouse Project (KOMP) and obtained from the KOMP Repository (www.komp.org). NIH grants to Velocigene at Regeneron Inc (U01HG004085) and the CSD Consortium (U01HG004080) funded the generation of gene-targeted ES cells for 8500 genes in the KOMP Program and archived and distributed by the KOMP Repository at UC Davis and CHORI (U42RR024244).

**Fig. 1:**
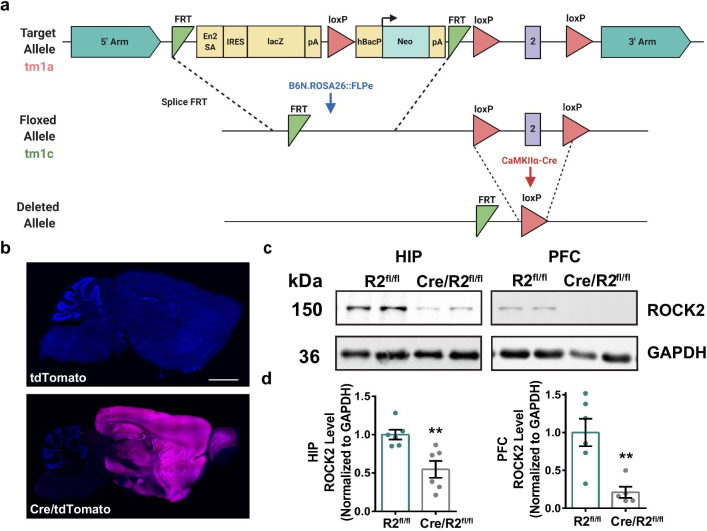
Generation of ROCK2^fl/fl^ mice. **a** Schematic representation of the initial allele (tm1a) containing LacZ reporter-promoter driven neo targeting cassette, FLP-FRT sites, loxP sites, and exon 2 that encodes for the kinase domain of ROCK2. The tm1a mice were crossed to B6N.ROSA26:FLPe mice to generate ROCK2 floxed mice (tm1c). Generation of floxed ROCK2 mice allows for *CaMKII*-Cre-mediated deletion of exon 2. **b** tdTomato signal in the forebrain of Cre/tdTomato compared to Cre-negative littermates. Scale bar = 2.5 mm. **c** Representative western blot of ROCK2 and GAPDH levels from hippocampus (HIP) and prefrontal cortex (PFC) of ROCK2^fl/fl^ and Cre/ROCK2^fl/fl^ mice at 8–9 months. **d** For densitometry analysis protein levels of whole homogenates from ROCK2^fl/fl^ and Cre/ROCK2^fl/fl^ were normalized to GAPDH. Cre/ROCK2^fl/fl^ whole homogenate showed decreased ROCK2 levels in the hippocampus (t(10) = 3.531, **p = 0.0054) and PFC (t(9) = 3.718, **p = 0.0048) compared to ROCK2^fl/fl^ mice. N = 6 ROCK2^fl/fl^ mice (3 M, 3 F) and 6 Cre/ROCK2^fl/fl^ (3 M, 3 F) mice at 8–9 months of age. Unpaired t-tests were used for all comparisons. ROUT outlier test identified one outlier in PFC data set (Cre/ROCK2^fl/fl^: 1.416336). Error bars represent standard error of the mean. R2^fl/fl^, ROCK2^fl/fl^; Cre/R2^fl/fl^, Cre/ROCK2^fl/fl^

To generate ROCK2^fl/fl^ mice, C57BL/6N-Rock2tm1a heterozygous mice were crossed to B6N.129S4-Gt(ROSA)26Sortm1(FLP1)Dym/J (JAX Stock No: 016226) to remove the KOMP selection cassette and generate C57BL/6N-Rock2tm1c mice (Fig. 1a). To remove the FLP1 transgene, C57BL/6N-Rock2tm1c heterozygous mice (ROCK2^fl/−^) were crossed with C57BL/6NJ (JAX Stock No: 005304) for two generations and absence of the FLP1 transgene was confirmed by PCR genotyping. Then, ROCK2^fl/−^ mice were interbred to generate ROCK2^fl/fl^ mice. ROCK2^fl/fl^ mice were crossed with B6.Cg-Tg(Camk2a-cre)T29-1Stl/J (JAX Stock No: 005359) (*CaMKII*-Cre) for one generation to generate Cre:ROCK2^fl/−^ mice, followed by interbreeding of offspring to yield Cre:ROCK2^fl/fl^ mice or ROCK2^fl/fl^ littermate controls (Fig. 1a). Henceforth, all experimental mice were on a mixed B6J; B6N genetic background. Mice were genotyped using DNA extracted from tail biopsies and PCR with gene-specific primers. All mice were 8–9 months old (unless otherwise indicated in the figure legend), and both males and females were used in each experiment. No statistically significant differences in sex were observed; therefore, all data is a combination of male and female mice (details are given in the figure legends). All experimenters were blind to genotypes until the conclusion of the study.

For experiments to evaluate the range of *CaMKII*-Cre expression in our colony, *CaMKII*-Cre mice, that were being used to mate with ROCK2^fl/fl^ mice, were crossed with B6.Cg-Gt(ROSA)26Sortm9(CAG-tdTomato)Hze/J (JAX Stock No: 007909). Offspring were used for experiments to observe the red fluorescent protein variant (tdTomato) and assess spread of Cre expression in the forebrain (Fig. 1b).

### Behavioral assessment

Mice were allowed to acclimate to the testing room for approximately 1 h prior to testing. All testing was performed at the same time each day within each cohort of animals. The apparatus was disinfected with 2% chlorhexidine prior to testing and cleaned with 70% ethanol between animals.

#### Elevated plus maze

The elevated plus maze apparatus (EPM; Med Associates) was 1 m high with 2 in wide arms. Two opposite arms had 8 in high black walls, while the other two opposing arms were open. Each mouse was placed one at a time in the center of the maze and the mouse was allowed to freely explore for 5 min. Exploration into arms was recorded and traced by the manufacturer’s software (CleverSys). Percent time in open arms was calculated by dividing the time in open arms by total time.

#### Light dark box test

The light dark box test was performed in a rectangular plexiglass box consisting of two compartments (one light, one dark). A removable dark partition was used to divide the box into a light and dark side. Mice were placed in the light compartment facing away from the dark partition and allowed to explore both compartments for 5 min. Activity was recorded and tracked with video tracking software (Cleversys).

#### Open field test

Mice were placed one at a time into a 16 in × 16 in plexiglass box (Med Associates) with opaque walls. Mice explored for 10 min. Ambulatory distance and ambulatory counts were determined by the manufacturer’s software (CleverSys).

#### Passive avoidance test

A 3-day passive avoidance protocol was employed. On the first day, mice were habituated to the apparatus, which consisted of two compartments (one light, one dark) separated by a guillotine door. Mice were placed in the illuminated compartment, with the guillotine door closed, and allowed to explore for 30 s. The guillotine door was opened, and latency to enter the dark compartment was recorded. An event was considered an entry only when all four paws were in the dark compartment, and at this point, the door was closed to confine the mouse to the dark compartment. On the second day, mice were again placed in the illuminated compartment for 30 s, after which the guillotine door was opened. Two seconds after entry into the dark compartment, mice received a 0.5 mA foot shock, which lasted for 2 s. After 30 s, mice were removed from the apparatus. Latency to enter the dark compartment was recorded. On the third day, mice were placed in the illuminated compartment. After 30 s, the guillotine door was opened. Latency to enter the dark compartment was recorded, with a maximum of 5 min. Any mice that did not enter the dark compartment were assigned a latency of 5 min.

### Perfusions and brain tissue processing

For iontophoretic microinjections, mice were anesthetized with Fatal Plus (Vortech Pharmaceuticals, Catalog #0298-9373-68) and transcardially perfused with cold 1% paraformaldehyde (PFA; Sigma Aldrich, Catalog #P6148) for 1 min, followed by 4% PFA with 0.125% glutaraldehyde (Fisher Scientific, Catalog #BP2547) for 10 min. A peristaltic pump (Cole Parmer) was used for consistent administration of cold PFA. Immediately following perfusion, each brain was extracted and drop-fixed in 4% PFA with 0.125% glutaraldehyde for 8–12 h at 4 °C. After fixation, brains were coronally sliced in 250 µm thick sections using a Leica vibratome (VT1000S, speed 70, frequency 7). Brains were sliced in 0.1 M PB and stored at 4 °C, one slice per well in a 48-well plate, in 0.1 M PB with 0.1% sodium azide (Fisher, Catalog #BP922I).

For tdTomato experiments, mice were anesthetized with Fatal Plus and transcardially perfused with cold 1× phosphate-buffered saline (PBS) for 2 min. Immediately following perfusion, each brain was extracted and dissected into two hemispheres. Both hemispheres were placed in 0.1 M PB with 0.1% sodium azide and stored at 4 °C. Right hemispheres were sliced in 50 µm thick sagittal sections using a Leica vibratome (VT1000S, speed 70, frequency 7). Brains were sliced in 0.1 M PB and stored at − 20 °C in cryoprotectant solution with 0.01% sodium azide (300 g sucrose, 500 mL 0.1 M phosphate buffer, 300 mL ethylene glycol, 0.1 g sodium azide, bring to 1 L with phosphate buffer). Sagittal sections were washed in 5 µL of 4ʹ,6-diamidino-2-phenylindole (DAPI) per 1.5 mL of 1× PBS for 15 min. Finally, slices were mounted on glass slides using Vectashield mounting medium (Vector Laboratories, Catalog#H-1000), sealed with clear nail polish, and stored at 4 °C.

For biochemistry, mice were anesthetized with Fatal Plus and transcardially perfused with cold 1× phosphate-buffered saline (PBS) for 2 min. Immediately following perfusion, each brain was extracted and dissected into two hemispheres. Both hemispheres were flash frozen in 2-methylbutane (Sigma, Catalog #320404), placed on dry ice, and stored at − 80 °C.

### Iontophoretic microinjections

Iontophoretic microinjections were performed based on methods described previously [27, 31]. A Nikon Eclipse FN1 upright microscope with a 10× objective and a 40× water objective was placed on an air table. The tissue chamber used consisted of a 50 × 75 mm plastic base with a 60 × 10 mm petri dish epoxied to the base. A platinum wire was attached so that the ground wire could be connected to the bath by an alligator clip. The negative terminal of the electric current source was connected to a glass micropipette filled with 2 µL of 8% Lucifer yellow dye (ThermoFisher, Catalog#L453). Micropipettes (A-M Systems, Catalog #603500) with highly tapered tips were pulled fresh at the time of use. A manual micromanipulator was secured on the air table with magnets that provided a 45° angle for injection. Brain slices were placed into a small petri dish containing 1× PBS and DAPI for 5 min at room temperature. After incubation in DAPI, slices were placed on dental wax, and then a piece of filter paper was used to adhere the tissue. The filter paper was then transferred to the tissue chamber filled with 1× PBS, and weighted down for stability. The 10× objective was used to visualize advancement of the tip of the micropipette in X, Y, and Z planes until the tip was just a few micrometers above the tissue. The 40× objective was then used while advancing the tip into layer 2/3 of the mPFC, dorsal and ventral CA1 regions of the hippocampus, or the basolateral amygdala. Once the microelectrode contacted a neuron, 2 nA of negative current were applied for 5 min to fill the neuron with Lucifer yellow. After 5 min, the current was turned off and the micropipette was removed from the neuron. Neuron impalement within the mPFC, CA1, and basolateral amygdala occurred randomly in a blind manner. If the entire neuron did not fill with dye after penetration, the electrode was removed and the neuron was not used for analysis. Multiple neurons were injected in each hemisphere of each animal. After injection, the filter paper containing the tissue was moved back into the chamber containing 1× PBS. The tissue was carefully lifted off the paper and placed on a glass slide with two 125 µm spacers (Electron Microscopy Sciences, Catalog #70327-20S). Excess PBS was carefully removed with a Kimwipe, and the tissue was air-dried for 1 min. One drop of Vectashield (Vector Labs, Catalog #H1000) was added directly to the slice, and the coverslip (Warner, Catalog #64-0716) was added and sealed with nail polish. Injected tissue was stored at 4 °C in the dark.

### Confocal microscopy

Confocal microscopy was used to capture images of Lucifer yellow-filled dendrites on layer 2/3 pyramidal neurons in the mPFC, pyramidal neurons in CA1 of the hippocampus, and amygdala neurons. Our methods are based on previously described methods [27, 31], and are detailed as follows. Imaging was performed by a blinded experimenter on a Nikon (Tokyo, Japan) Ti2 C2 confocal microscope, using a Plan Apo 60×/1.40 NA oil-immersion objective. Three-dimensional z-stacks were obtained of secondary dendrites from dye-impregnated neurons that met the following criteria: (1) within 80 µm working distance of microscope, (2) relatively parallel with the surface of the coronal section, (3) no overlap with other branches, (4) located between 40 and 120 µm from the soma. Nikon Elements 4.20.02 image capture software was used to acquire z-stacks with a step size of 0.1 µm, image size of 1024 × 512 px, zoom of 4.8×, line averaging of 4×, and acquisition rate of 1 frame/s.

Confocal microscopy was also used to capture images of slices from Cre/tdTomato and Cre-negative/tdTomato littermates. Imaging was performed on a Nikon Ti2 C2 confocal microscope, using a Plan Fluor 10×/0.75 NA air objective. Nikon Elements 4.20.02 image capture software was used to acquire stitched images (20 × 10 field) by automatically stitching multiple adjacent 10× images using 25% blending overlap. Each frame had an image size of 1024 × 1024 px, and acquisition rate of 1 frame/s. Subsequently, 10× stitched images were acquired, with all other imaging parameters remaining the same. Following image acquisition, post-image processing was performed using Fiji ImageJ. Each image file was a two channel image. The Split Channels function was used to separate the two channels. Brightness was adjusted for each channel separately, and then the Merge Channels function was used to overlay the DAPI and TRITC images.

### Dendritic spine morphometry analysis

Confocal z-stacks of dendrites were deconvolved using Huygens Deconvolution System (16.05, Scientific Volume Imaging, the Netherlands). The following settings were used: deconvolution algorithm: GMLE; maximum iterations: 10; signal to noise ratio: 15; quality: 0.003. Deconvolved images were saved in .tif format. Deconvolved image stacks were imported into Neurolucida 360 (2.70.1, MBF Biosciences, Williston, Vermont) for dendritic spine analysis. For each image, the semi-automatic directional kernel algorithm was used to trace the dendrite. The outer 5 µm of each dendrite were excluded from the trace. All assigned points were examined to ensure they matched the dendrite diameter and position in X, Y, and Z planes, and were adjusted if necessary. Dendritic spine reconstruction was performed automatically using a voxel-clustering algorithm, with the following parameters: outer range = 5 µm, minimum height = 0.3 µm, detector sensitivity = 80%, minimum count = 8 voxels. The semi-automatically identified spines were examined to ensure that all identified spines were real, and that all existing spines had been identified. If necessary, spines were added by increasing the detector sensitivity. Additionally, merge and slice tools were used to correct errors made in the morphology and backbone points of each spine. Each dendritic spine was automatically classified as a dendritic filopodium, thin spine, stubby spine, or mushroom spine based on constant parameters [27, 31]. Three-dimensional dendrite reconstructions were exported to Neurolucida Explorer (2.70.1, MBF Biosciences, Williston, Vermont) for branched structure analysis, which provides measurements of dendrite length; number of spines; spine length; number of thin, stubby, and mushroom spines, and filopodia; spine head diameter; and spine neck diameter, among other measurements. The output was exported to Microsoft Excel (Redmond, WA). Spine density was calculated as the number of spines per 10 µm of dendrite length. Values per mouse were calculated by averaging the values for all dendrites corresponding to that mouse. To assess apical and basal dendrites separately, values for either apical or basal dendrites were averaged per mouse. For a mouse to be included in statistical analyses for dendritic spine density and morphology a minimum of 100 µm of dendrite length had to be reconstructed and analyzed. The number of neurons, dendrites, dendritic length, and spines analyzed per animal are provided in Additional file 1: Table S1.

### Biochemical fractionation and western blotting

Flash-frozen hemispheres were sub-dissected to obtain the prefrontal cortex and hippocampus. Samples were thawed in 75 µL of ice cold TEVP buffer + 320 mM sucrose and homogenized. The homogenate was centrifuged for 10 min at 800×*g* at 4 °C. The supernatant was saved as a whole homogenate and stored at − 80 °C. Protein concentrations were determined by BCA protein assay (Pierce). GAPDH-enriched and PSD95-enriched fractionations were performed, based on previously described methods [34]. Samples were homogenized in 75 µL of sucrose buffer and centrifuged at 3000×*g* at 4 °C. The resulting supernatant was collected and centrifuged at 10,000×*g* at 4 °C. Following centrifugation, the supernatant was saved as the GAPDH-enriched fraction. The remaining pellet was washed in 50 µL of sucrose buffer, resuspended in 60 µL of HBS with 2% Triton X-100 and incubated on ice for 30 min. Followed by, centrifugation at 10,000×*g* for 20 min at 4 °C. The remaining supernatant was saved as the synaptophysin-enriched fraction and the pellet was resuspended in 30 µL of 1× PBS and saved as the PSD95-enriched fraction. Twenty-five micrograms of protein per sample were loaded into each well for SDS-PAGE. Primary antibodies were incubated overnight at 4 °C. Secondary antibodies were incubated for 1 h at room temperature. Images were captured using an Odyssey Image Station (Li-Cor), and band intensities were quantified using Odyssey Application Software (3.0, Li-Cor). Primary antibodies: ROCK2 (D1B1) Rabbit mAb (Cell Signaling, Catalog #9029S), Glyceraldehyde-3-Phosphate Dehydrogenase (GAPDH) (6C5) Mouse mAb (MilliporeSigma, Catalog #MAB374), Synaptophysin (D35E4) Rabbit mAB (Cell Signaling, Catalog #5461), PSD-95 (D27E11) Rabbit mAb (Cell Signaling, Catalog #3450), Phospho-mTOR (Ser2448) Rabbit mAb (Cell Signaling, Catalog #2971S), mTOR Rabbit mAb (Cell Signaling, Catalog #2972S), AMPA Receptor 1 Rabbit mAb (Cell Signaling, Catalog #13185), Phospho-LIMK Rabbit mAb (Cell Signaling, Catalog #3841), SQSTM1/p62 Rabbit mAb (Cell Signaling, Catalog #5114S), NMDA Receptor 1 Rabbit mAb (Cell Signaling, Catalog #5704), and α-tubulin Mouse mAb clone #12G010 (Developmental Studies Hybridoma Bank, University of Iowa). Secondary antibody: AlexaFluor 680 goat anti-rabbit (Life Technologies, Catalog #A21109) and AlexaFluor 488 donkey anti-mouse IgG (H + L) (Life Technologies, Catalog #A21202).

### Statistical analysis

Statistical analyses were conducted using GraphPad Prism 8.4.0 (GraphPad Software, La Jolla, CA). Data are presented as mean ± SEM, and all graph error bars represent SEM. Assuming Gaussian distribution, two-tailed unpaired t*-*tests with a 95% confidence interval were used for comparison of biochemistry, behavior, and dendritic spine morphometry between ROCK2^fl/fl^ and Cre/ROCK2^fl/fl^ mice. Significance was defined as p < 0.05. ROUT’s method with a false discovery rate specified to 5% was used to identify outliers in biochemistry analysis. To compare spine densities or morphologies among experimental conditions, the mean spine density or morphologic measurement was calculated per experimental replicate (or *N*). These experiment means were then averaged per experimental condition and reported as a condition mean. See figure legends for details on *N* per experiment.

## Results

### ROCK2 deficiency induces anxiety-like behaviors

ROCK2^fl/fl^ mice were generated from C57BL/6N-Rock2^tm1a(KOMP)Wtsi^ ES cells that were purchased from the NIH Knock-Out Mouse Project (KOMP) and obtained from the KOMP Repository. C57BL/6N-Rock2^tm1a^ heterozygous mice were crossed to B6N.129S4-Gt(ROSA)26Sor^tm1(FLP1)Dym/J^ that have wide-spread expression of flippase recombinase to generate C57BL/6N-Rock2^tm1c^ heterozygous mice (ROCK2^fl/−^) (Fig. 1a). ROCK2^fl/fl^ mice harbor loxP sites that flank the critical exon 2 that encodes the ROCK2 kinase domain. To eliminate ROCK2 from forebrain excitatory neurons, ROCK2^fl/fl^ mice were crossed with B6.Cg-Tg(Camk2a-cre)T29-1Stl/J mice (*CaMKII*-Cre) to yield experimental animals that are positive for Cre recombinase (Cre/ROCK2^fl/fl^) or ROCK2^fl/fl^ littermate controls that are negative for Cre recombinase (Fig. 1a) [35]. To test *CaMKII*-Cre expression in our colony, *CaMKII*-Cre breeders that were being used to mate with ROCK2^fl/fl^ mice were crossed with B6.Cg-Gt(ROSA)26Sor^tm9(CAG-tdTomato)Hze/J^ mice (Cre/tdTomato). Offspring were evaluated for expression of a red fluorescent protein variant (tdTomato) using confocal microscopy. Cre/tdTomato mice displayed robust tdTomato signal in the forebrain, including the prelimbic prefrontal cortex, hippocampus, and amygdala, compared to Cre-negative littermates (Fig. 1b). Next, we examined ROCK2 protein levels in the hippocampus and prelimbic prefrontal cortex of Cre/ROCK2^fl/fl^ mice using biochemical methods (Fig. 1c). Densitometry analysis of western blots indicated that ROCK2 protein levels were reduced significantly in the hippocampus and PFC of Cre/ROCK2^fl/fl^ compared to ROCK2^fl/fl^ littermate controls (Fig. 1d). While the level of reduction of ROCK2 protein in the hippocampus and PFC seems to indicate that excitatory neurons are the primary cell type that expresses ROCK2, RNA-sequencing transcriptome and splicing data indicates that non-neuronal cell types express relatively similar levels of ROCK2 as neurons [36]. However, there may be differences in ROCK2 protein level due to cell type specific translation of ROCK2 transcript [37].

**Fig. 2:**
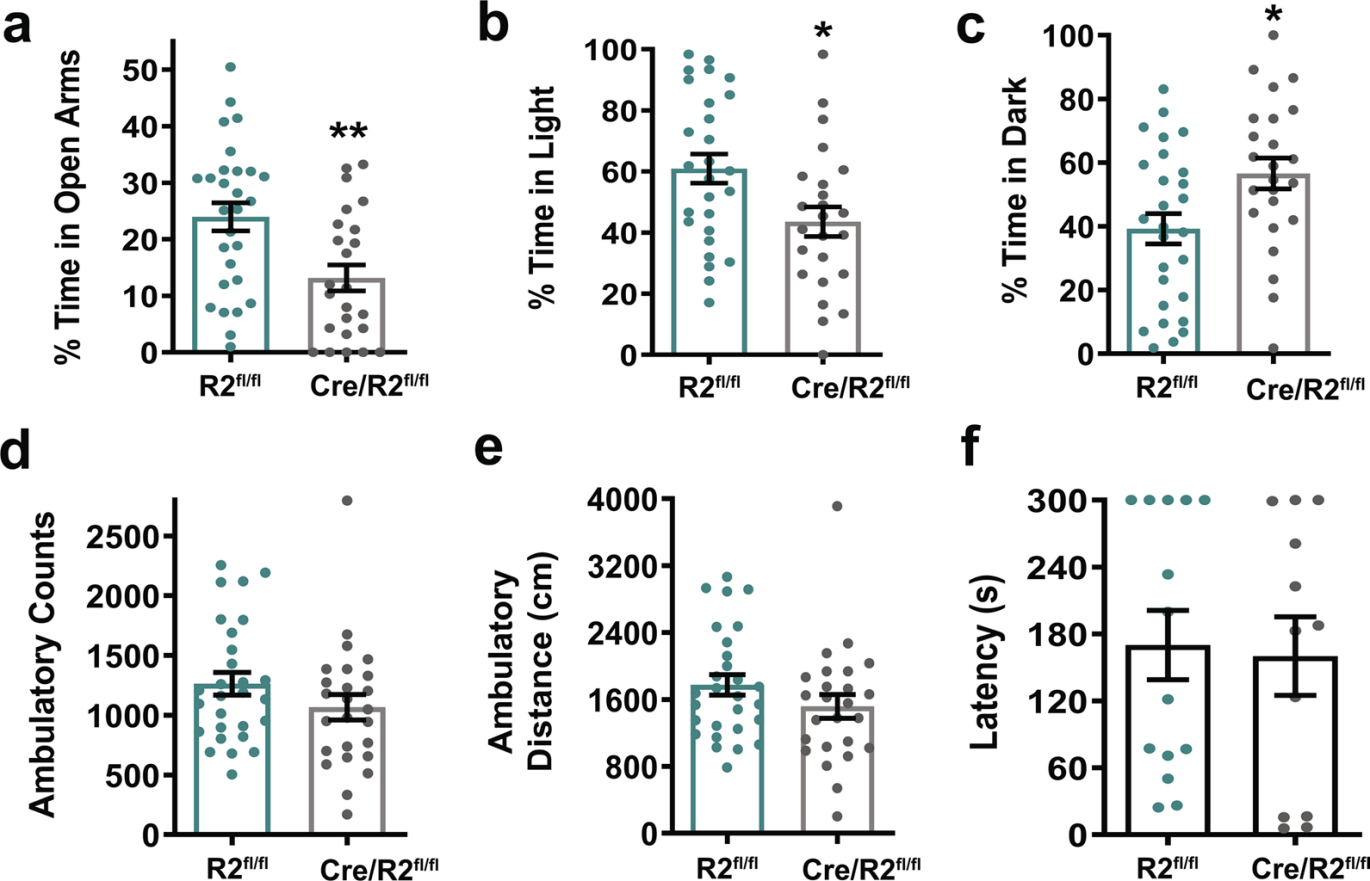
Cre/ROCK2^fl/fl^ mice exhibit anxiety-like behaviors. **a** Cre/ROCK2^fl/fl^ mice spent less time in the open arms of the elevated plus maze (EPM) compared to ROCK2^fl/fl^ (t(50) = 3.140, **p = 0.0028). N = 28 ROCK2^fl/fl^ mice (14 M, 14 F) and 24 Cre/ROCK2^fl/fl^ (11 M, 13 F). **b**, **c** Light Dark Box Test. **b** Cre/ROCK2^fl/fl^ mice spent less time in the light box compared to ROCK2^fl/fl^ mice (t(49) = 2.557, *p = 0.0137) and **c** more time in the dark box (t(49) = 2.562, *p = 0.0135). N = 27 ROCK2^fl/fl^ mice (13 M, 14 F) and 24 Cre/ROCK2^fl/fl^ (11 M, 13 F). **d**, **e** Open field test for ROCK2^fl/fl^ (N = 28: 14 males, 14 females) and Cre/ROCK2^fl/fl^ (N = 25: 12 M, 13 F) mice. **d** Ambulatory counts and **e** ambulatory distance were measured. **f** Cre/ROCK2^fl/fl^ mice did not show any changes in latency to enter the dark compartment in the passive avoidance test compared to ROCK2^fl/fl^ mice. N = 14 ROCK2^fl/fl^ mice (7 M, 7 F) and 12 Cre/ROCK2^fl/fl^ (6 M, 6 F) mice at 8–9 months of age. Unpaired t-tests were used for all comparisons. Each point represents one mouse and error bars represent standard error of the mean. R2^fl/fl^, ROCK2^fl/fl^; Cre/R2^fl/fl^, Cre/ROCK2^fl/fl^

The elevated plus maze (EPM) was used to assess potential anxiety-like behaviors [38]. The amount of time spent in the open arms of the EPM was reduced significantly in Cre/ROCK2^fl/fl^ mice compared to ROCK2^fl/fl^ littermates (Fig. 2a). As a secondary assessment of anxiety-like behaviors, the light–dark box test was performed [39]. Cre/ROCK2^fl/fl^ mice spent a significantly greater percent of time in the dark side and significantly less time in the light side of the light–dark box compared to ROCK2^fl/fl^ littermates (Fig. 2b, c). Results from the open field test indicated that locomotor activity was comparable in both genotypes as measured by ambulatory counts and distance (Fig. 2d, e). To evaluate cognitive abilities involving the hippocampus and prelimbic prefrontal cortex, we performed the passive avoidance test [40]. Latency to enter the dark chamber was comparable between genotypes on Day 3 of the test, suggesting that the Cre/ROCK2^fl/fl^ mice did not display learning and memory dysfunction (Fig. 2f). Collectively, these results indicate that Cre/ROCK2^fl/fl^ mice exhibit anxiety-like behavior.

### ROCK2 deficiency alters spine density and volume in CA1

**Fig. 3:**
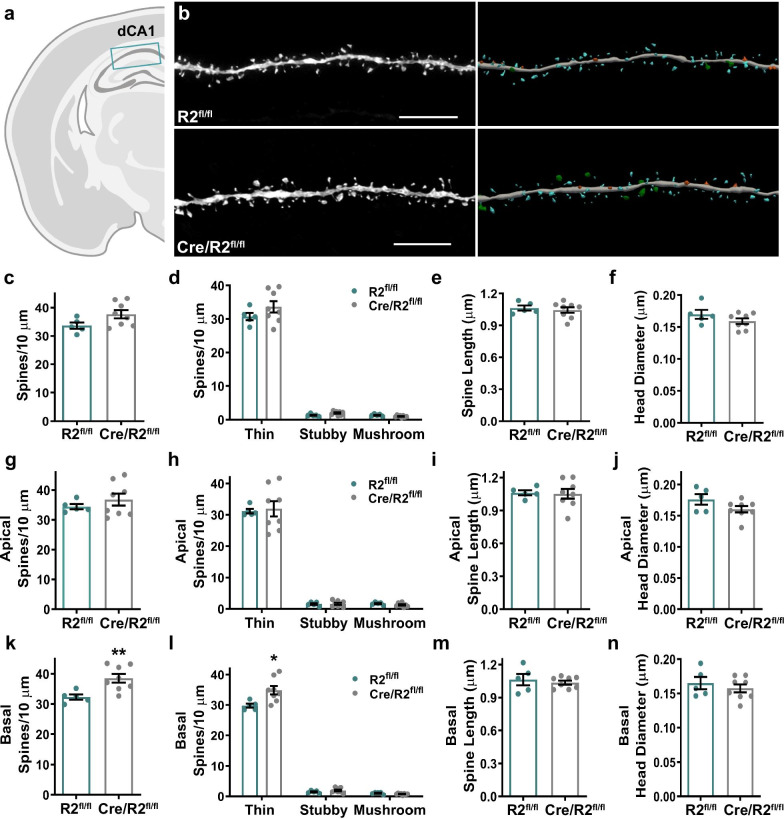
Cre/ROCK2^fl/fl^ mice display increased basal spine density among dorsal CA1 pyramidal neurons. **a** Schematic representation of iontophoretic injection sites in dorsal CA1 (dCA1) of the hippocampus. **b** Representative maximum intensity projections of deconvolved confocal z-stacks of Lucifer yellow-filled dendrites of dCA1 pyramidal neurons are shown for ROCK2^fl/fl^ and Cre/ROCK2^fl/fl^ mice (left). Corresponding three-dimensional reconstructions of the dendrites are provided (right), with the spines color-coded by spine type (blue = thin, orange = stubby, green = mushroom, yellow = filopodia). Scale bars = 6 µm. **c** There was no difference in overall mean spine density and **d** densities of thin, stubby, or mushroom spines between groups. Overall mean of **e** spine length and **f** head diameter were similar between groups. **g**–**j** Apical **g** spine density, **h** densities of thin, stubby, or mushroom spines, **i** spine length, and **j** head diameter were similar between groups. **k** Cre/ROCK2^fl/fl^ mice show increased basal spine density (t(11) = 3.162, **p = 0.0091) compared to ROCK2^fl/fl^ mice. **l** Basal thin spines density was increased in Cre/ROCK2^fl/fl^ (t(11) = 2.759, *p = 0.0186) compared to ROCK2^fl/fl^ littermate controls. **m**, **n** There was no difference in basal spine length (**m**) and head diameter (**n**) between groups. N = 5 ROCK2^fl/fl^ mice (1 M, 4 F) and 8 Cre/ROCK2^fl/fl^ (5 M, 3 F) mice at 8–9 months of age. Unpaired t-tests were used for all comparisons. Each point represents one mouse and the error bars indicate the standard error of the mean. R2^fl/fl^, ROCK2^fl/fl^; Cre/R2^fl/fl^, Cre/ROCK2^fl/fl^

Several brain regions, including the hippocampus, mPFC, and amygdala, can be involved with the exhibition of anxiety-like behaviors in the EPM [33, 41]. To test whether ROCK2 reduction in excitatory neurons alters spine density and morphology in the hippocampus, an iontophoretic fluorescent dye loading strategy was employed to visualize single neurons in the dorsal and ventral CA1 regions. First, lucifer yellow was delivered intracellularly to pyramidal neurons within dorsal CA1 (dCA1) (Fig. 3a). Raw confocal images were subjected to deconvolution and then morphometric analyzation of dendritic spines was performed (Fig. 3b). Additional file 1: Table S1 lists the N for spines, dendrites, and mice as well as total dendritic length that was measured for each brain region. There were no significant differences among dCA1 pyramidal neurons in mean spine density between Cre/ROCK2^fl/fl^ and ROCK2^fl/fl^ mice (Fig. 3c). Moreover, densities of spine subtypes were comparable between Cre/ROCK2^fl/fl^ and ROCK2^fl/fl^ mice (Fig. 3d). Mean spine length as well as mean spine head diameter was similar between Cre/ROCK2^fl/fl^ and ROCK2^fl/fl^ mice (Fig. 3e, f). Next, spines were separated based on whether they were sampled from apical or basal dendrites. There was no significant difference in mean density, spine subtype densities, mean length, or mean head diameter on apical dendrites (Fig. 3g–j). However, overall mean spine density on basal dendrites was increased significantly in Cre/ROCK2^fl/fl^ mice compared to ROCK2^fl/fl^ littermates, and this was driven by a significant increase in thin spine density (Fig. 3k, l). Mean spine length and mean head diameter were similar between genotypes on basal dendrites (Fig. 3m, n).

**Fig. 4:**
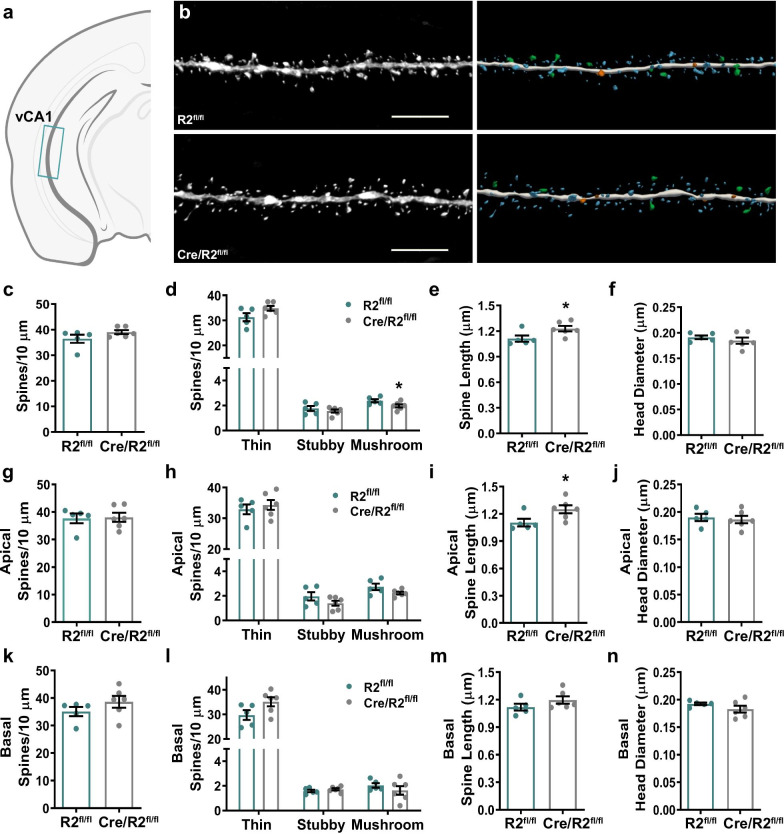
Cre/ROCK2^fl/fl^ mice display increased apical spine length among ventral CA1 pyramidal neurons. **a** Schematic representation of iontophoretic injection sites in ventral CA1 (vCA1) of the hippocampus. **b** Representative maximum intensity projections of deconvolved confocal z-stacks of Lucifer yellow-filled dendrites in vCA1 are shown (left) for ROCK2^fl/fl^ and Cre/ROCK2^fl/fl^ mice. Corresponding three-dimensional reconstructions of the dendrites are provided (right), with the spines color-coded by spine type (blue = thin, orange = stubby, green = mushroom, yellow = filopodia). Scale bars = 6 µm. **c** Overall mean spine density and **d** densities of thin and stubby were similar between groups yet, Cre/ROCK2^fl/fl^ mice showed decreased mushroom spine density (t(9) = 1.975, *p = 0.0462) compared to ROCK2^fl/fl^ littermates. **e** Cre/ROCK2^fl/fl^ mice show increased mean spine length (t(9) = 2.367, *p = 0.0421) compared to ROCK2^fl/fl^ mice. **f** There were no differences in overall mean head diameter between groups. There were no differences in **g** apical spine density and **h** densities of thin, stubby, and mushroom spines. **i** Apical spine length was increased in Cre/ROCK2^fl/fl^ mice (t(9) = 2.376, *p = 0.0415) compared to ROCK2^fl/fl^ littermates. **j** There were no differences in apical head diameter between groups. Cre/ROCK2^fl/fl^ mice had no differences in basal **k** spine density, **l** densities of thin, stubby, and mushroom spines, **m** spine length, and **n** head diameter compared to ROCK2^fl/fl^ littermates. N = 5 ROCK2^fl/fl^ mice (2 M, 3 F) and 6 Cre/ROCK2^fl/fl^ (3 M, 3 F) mice at 8–9 months of age. Unpaired t-tests were used for all comparisons. Each point represents one mouse and the error bars indicate the standard error of the mean. R2^fl/fl^, ROCK2^fl/fl^; Cre/R2^fl/fl^, Cre/ROCK2^fl/fl^

To test whether Cre/ROCK2^fl/fl^ mice exhibited alterations of spine density and morphology within ventral CA1 (vCA1) (Fig. 4a), individual pyramidal neurons were targeted for iontophoretic microinjection of Lucifer yellow, followed by high-resolution confocal laser scanning microscopy and dendritic spine morphometry analysis (Fig. 4b). Cre/ROCK2^fl/fl^ and ROCK2^fl/fl^ mice exhibited no significant differences in mean spine density among vCA1 pyramidal neurons (Fig. 4c). Densities of spine subtypes were mostly comparable between Cre/ROCK2^fl/fl^ and ROCK2^fl/fl^ mice, however there was a subtle but significant reduction in mushroom spine density in Cre/ROCK2^fl/fl^ mice (Fig. 4d). Overall mean spine length among vCA1 pyramidal neurons was significantly increased in Cre/ROCK2^fl/fl^ mice compared to ROCK2^fl/fl^ littermates (Fig. 4e). Overall mean head diameter was similar between genotypes (Fig. 4f). The increase in overall mean spine length among Cre/ROCK2^fl/fl^ mice was driven by a significant increase in spine length on apical, but not basal, dendrites; however, there was no significant difference in mean density, spine subtype densities, or mean head diameter among apical or basal dendrites (Fig. 4g–n).

**Fig. 5:**
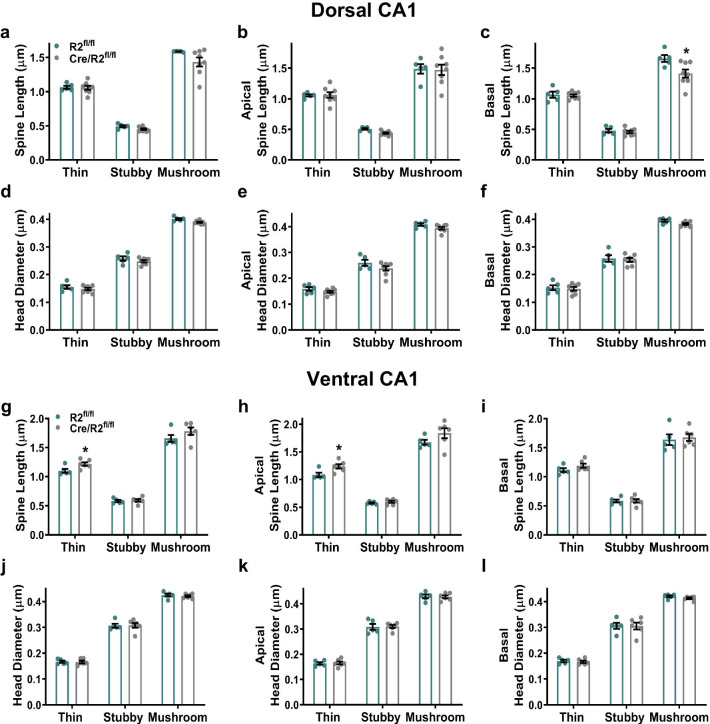
Cre/ROCK2^fl/fl^ mice display changes in spine length among spine subclasses on CA1 pyramidal neurons. Dorsal CA1 (dCA1): **a** There were no differences in mean spine length of thin, stubby, or mushroom spines between groups. **b** The lengths of apical spine subclasses were similar between groups. **c** Basal mushroom spine length was decreased in Cre/ROCK2^fl/fl^ mice (t(11) = 2.563, *p = 0.0264) compared to ROCK2^fl/fl^ mice. **d**, **f** Head diameter was similar between groups for **d** all dendrites combined, or **e** apical or **f** basal dendrites. Ventral CA1 (vCA1): **g** Mean thin spine length was increased in Cre/ROCK2^fl/fl^ mice (t(9) = 2.600, *p = 0.0287) compared to ROCK2^fl/fl^ littermates. **h** Cre/ROCK2^fl/fl^ mice had increased apical thin spine length (t(9) = 2.707, *p = 0.0241) compared to ROCK2^fl/fl^ littermates. **i** There were no differences in basal spine lengths of thin, stubby, or mushroom spines between groups. **j**, **l** There were no differences in head diameter for **j** all dendrites combined, or **k** apical or **l** basal spines between groups. dCA1: N = 5 ROCK2^fl/fl^ mice (1 M, 4 F) and 8 Cre/ROCK2^fl/fl^ (5 M, 3 F) mice. vCA1: N = 5 ROCK2^fl/fl^ mice (2 M, 3 F) and 6 Cre/ROCK2^fl/fl^ (3 M, 3 F) mice at 8–9 months of age. Unpaired t-tests were used for all comparisons. Each point represents one mouse and the error bars indicate the standard error of the mean. R2^fl/fl^, ROCK2^fl/fl^; Cre/R2^fl/fl^, Cre/ROCK2^fl/fl^

Further investigation of spine length among spine subclasses on apical or basal dendrites revealed that Cre/ROCK2^fl/fl^ mice exhibited significantly reduced basal mushroom spine length compared to ROCK2^fl/fl^ mice among dCA1 neurons (Fig. 5a–c). Spine head diameter among spine subclasses were similar between genotypes on apical and basal dendrites of dCA1 neurons (Fig. 5d–f). Thin spine length was increased significantly on vCA1 neurons in Cre/ROCK2^fl/fl^ mice, and these observations were driven by increases in thin spine length on apical, but not basal, dendrites (Fig. 5g–i). No changes in spine head diameter were observed among spine subclasses between genotypes on apical and basal dendrites of vCA1 neurons (Fig. 5j–l).

**Fig. 6:**
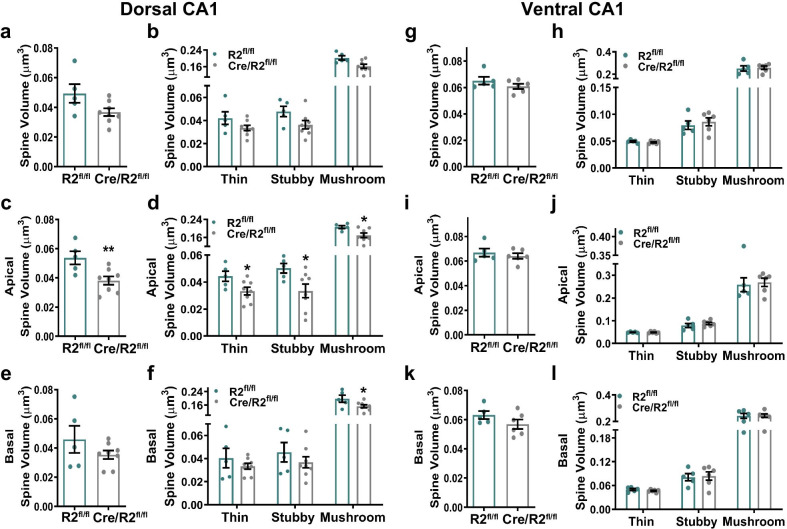
Cre/ROCK2^fl/fl^ mice exhibit reduced apical spine head volume among dorsal CA1 pyramidal neurons. Dorsal CA1 (dCA1): **a** Spine volume of all dendrites combined was similar between groups. **b** There were no differences in the volume of thin, stubby, or mushroom spines between groups. **c** Cre/ROCK2^fl/fl^ mice show decreased apical spine volume (t(11) = 3.127, **p = 0.0096) compared to ROCK2^fl/fl^ mice. **d** There were decreased thin (t(11) = 2.348, *p = 0.0386), stubby (t(11) = 2.389, *p = 0.0359), and mushroom (t(11) = 2.627, *p = 0.0235) spine volume in Cre/ROCK2^fl/fl^ compared to ROCK2^fl/fl^ mice. **e** There was no difference in basal spine volume between groups. **f** There were no differences in the volume of basal thin and stubby spines between groups but, Cre/ROCK2^fl/fl^ mice had reduced mushroom spine volume (t(11) = 2.219, *p = 0.0485) compared to ROCK2^fl/fl^ mice. Ventral CA1 (vCA1): Cre/ROCK2^fl/fl^ mice had similar **g** overall spine volume and **h** spine volume of thin, stubby, or mushroom subclasses compared to ROCK2^fl/fl^ mice. There were no differences in overall **i** apical spine volume, **j** spine subclasses or **k** basal spine volume or volume of **l** basal spine subclasses. dCA1: N = 5 ROCK2^fl/fl^ mice (1 M, 4 F) and 8 Cre/ROCK2^fl/fl^ (5 M, 3 F) mice; vCA1: N = 5 ROCK2^fl/fl^ mice (2 M, 3 F) and 7 Cre/ROCK2^fl/fl^ (4 M, 3 F) mice at 8–9 months of age. Unpaired t-tests were used for all comparisons. Each point represents one mouse and the error bars indicate the standard error of the mean. R2^fl/fl^, ROCK2^fl/fl^; Cre/R2^fl/fl^, Cre/ROCK2^fl/fl^

Next, we measured dendritic spine head volume among dCA1 pyramidal neurons. While overall spine head volume, including spine subclasses, was not significantly different between genotypes, spine head volume was reduced significantly on apical dendrites from Cre/ROCK2^fl/fl^ mice (Fig. 6a–c). Significant reductions in volume were observed among all spine subclasses on apical dendrites from Cre/ROCK2^fl/fl^ mice (Fig. 6d). Volume was significantly reduced only among mushroom spines on basal dendrites of Cre/ROCK2^fl/fl^ mice (Fig. 6e, f). There were no significant differences in overall spine head volume, among spine subclasses or basal or apical dendrites on vCA1 neurons between Cre/ROCK2^fl/fl^ and ROCK2^fl/fl^ littermates (Fig. 6g–j). Together, these results indicated that ROCK2 deficiency in dCA1 pyramidal neurons results in a decrease in spine head volume on apical dendrites, but an increase of thin spine density with reduced mushroom spine length on basal dendrites. On vCA1 pyramidal neurons, ROCK2 deficiency results in substantial increases in spine length, especially among thin spines on apical dendrites. Our findings raise the possibility that ROCK2 differentially regulates dendritic spine structural plasticity in dorsal and ventral CA1 excitatory neurons of the hippocampus.

Dendritic spine head volume is correlated with the size of the postsynaptic density, and specifically the abundance of *N*-methyl-d-aspartate (NMDA) and α-amino-3-hydroxy-5-methyl-4-isoxazolepropionic acid (AMPA) receptors that can drive overall synaptic strength [42–45]. Therefore, we hypothesized that the reduction in spine head volume that was observed in Cre/ROCK2^fl/fl^ mice may reflect lower levels of NMDA and AMPA receptors in the hippocampus. To test this hypothesis, we prepared GAPDH-enriched and PSD95-enriched fractionations from hippocampal homogenates using biochemical methods, then assessed protein levels of specific subunits glutamate receptor 1 (GluA1) of AMPA receptors and glutamate receptor 1 (GluN1) of NMDA receptors with western blots. Densitometry analysis indicated that GluA1 and GluN1 protein levels were comparable in GAPDH-enriched and PSD95-enriched fractionations, respectively, from Cre/ROCK2^fl/fl^ mice and ROCK2^fl/fl^ littermate controls (Additional file 2: Fig. S1). Although it is challenging to interpret, the comparative lack of change in GluN1 or GluA1 protein levels among Cre/ROCK2^fl/fl^ mice may reflect the increase in spine density on basal arbors in dCA1, which compensates for the reduced spine head volume on apical arbors.

### Spine density and morphology in the mPFC and amygdala are not altered by ROCK2 deficiency

**Fig. 7:**
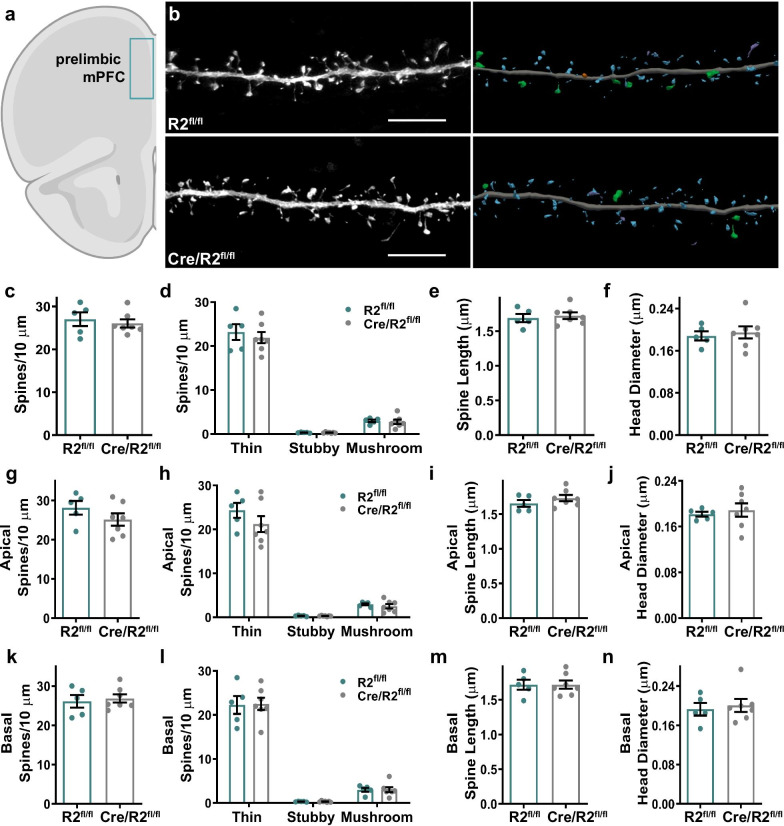
Medial prefrontal cortex spine density and morphology in Cre/ROCK2^fl/fl^ mice. **a** Schematic representation of iontophoretic injection sites in layers 2/3 of the prelimbic medial prefrontal cortex (mPFC). **b** Representative maximum intensity projections of deconvolved confocal z-stacks of Lucifer yellow-filled dendrites in layer 2/3 medial prefrontal cortex are shown (left) for ROCK2^fl/fl^ and Cre/ROCK2^fl/fl^ mice at 8–9 months of age. Corresponding three-dimensional reconstructions of the dendrites are provided (right), with the spines color-coded by spine type (blue = thin, orange = stubby, green = mushroom, yellow = filopodia). Scale bars = 6 µm. **c** Overall mean spine density and **d** densities of thin, stubby, and mushroom spines, **e** spine length, and **f** head diameter were similar between groups. There were no differences in apical **g** spine density, **h** densities of thin, stubby, and mushroom spines, **i** spine length, and **j** head diameter. Cre/ROCK2^fl/fl^ mice had no differences in basal **k** spine density, **l** densities of thin, stubby, and mushroom spines, **m** spine length, and **n** head diameter compared to ROCK2^fl/fl^ littermates. N = 5 ROCK2^fl/fl^ mice (2 M, 3 F) and 7 Cre/ROCK2^fl/fl^ (4 M, 3 F) mice at 8–9 months of age. Unpaired t-tests were used for all comparisons. Each point represents one mouse and the error bars indicate the standard error of the mean. R2^fl/fl^, ROCK2^fl/fl^; Cre/R2^fl/fl^, Cre/ROCK2^fl/fl^

The prelimbic mPFC is critical for normal anxiety-related behaviors in the EPM [46], and mouse avoidance of the open arms is linked to alterations in the firing rate of single neurons in the mPFC [33]. To test whether Cre/ROCK2^fl/fl^ mice exhibit dendritic spine changes in the prelimbic mPFC (Fig. 7a), individual pyramidal neurons in layers 2/3 were targeted for iontophoretic microinjection of Lucifer yellow, followed by high-resolution confocal laser scanning microscopy and dendritic three-dimensional reconstructions (Fig. 7b). ROCK2^fl/fl^ and Cre/ROCK2^fl/fl^ mice exhibited similar overall mean spine density, including spine subclasses, in the mPFC (Fig. 7c, d). Spine length and head diameter were comparable between genotypes, and this trend continued when spines were analyzed from apical or basal dendrites as well as among spine subclasses (Fig. 7e–n and Additional file 2: Fig. S2). Dendritic spine head volume was also measured, however no significant differences were observed between ROCK2^fl/fl^ and Cre/ROCK2^fl/fl^ mice (Additional file 2: Fig. S3).

**Fig. 8:**
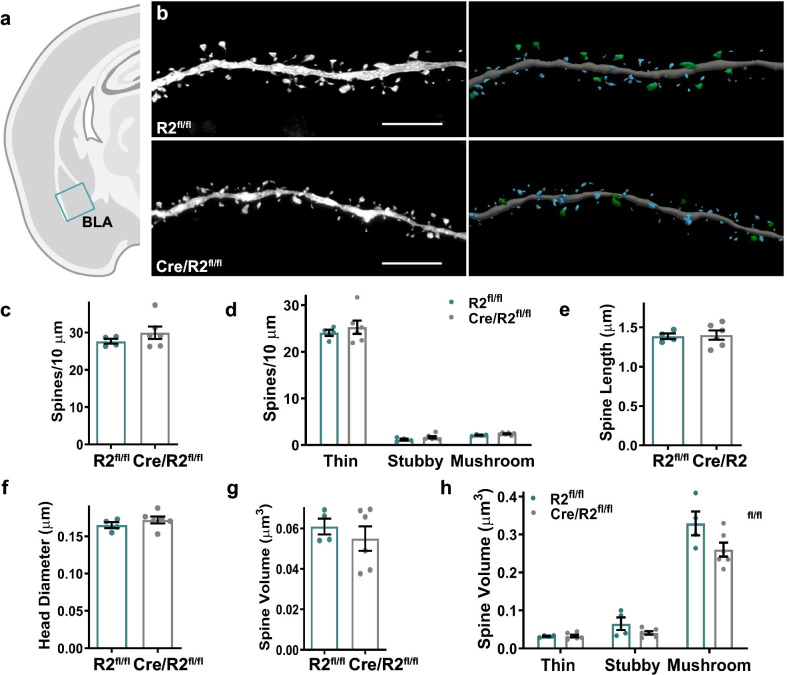
Basolateral amygdala spine density and morphology in Cre/ROCK2^fl/fl^ mice. **a** Schematic representation of iontophoretic injection sites in the basolateral amygdala (BLA). **b** Representative maximum intensity projections of deconvolved confocal z-stacks of Lucifer yellow-filled dendrites in basolateral amygdala are shown (left) for ROCK2^fl/fl^ and Cre/ROCK2^fl/fl^ mice at 8–9 months of age. Corresponding three-dimensional reconstructions of the dendrites are provided (right), with the spines color-coded by spine type (blue = thin, orange = stubby, green = mushroom, yellow = filopodia). Scale bars = 6 µm. Cumulative mean **c** spine density and **d** densities of thin, stubby, and mushroom spines, **e** spine length, and **f** head diameter of Cre/ROCK2^fl/fl^ mice were similar compared to ROCK2^fl/fl^ mice. There were no differences in **g** mean spine volume and **h** spine volume among spine subclasses. N = 4 ROCK2^fl/fl^ mice (1 M, 3 F) and 6 Cre/ROCK2^fl/fl^ (4 M, 2 F) mice at 8–9 months of age. Unpaired t-tests were used for all comparisons. Each point represents one mouse and the error bars indicate the standard error of the mean. R2^fl/fl^, ROCK2^fl/fl^; Cre/R2^fl/fl^, Cre/ROCK2^fl/fl^

The basolateral amygdala (BLA) is a critical region in the neural circuitry that mediates anxiety-related behaviors [47–49]. Moreover, experiments involving freely behaving mice demonstrated that groups of neurons in the BLA fire under anxiety-like conditions in the EPM [49]. Therefore, individual pyramidal neurons in the BLA were targeted for iontophoretic microinjection of Lucifer yellow (Fig. 8a), followed by high-resolution confocal laser scanning microscopy and dendritic spine morphometry analysis (Fig. 8b). Due to the complex nature of cell orientation and density within the BLA, we were unable to assess whether dendritic spine changes existed on polar dendritic arbors of pyramidal neurons. Henceforth, spine data from the BLA is a combination of analysis from apical and basal dendrites (Additional file 1: Table S1). Similar to findings in the mPFC, ROCK2^fl/fl^ and Cre/ROCK2^fl/fl^ mice displayed similar mean spine density, including spine subclasses, in the BLA (Fig. 8c, d). Spine morphology, including mean length and mean head diameter, were comparable between genotypes as well as among spine subclasses (Fig. 8e, f, and Additional file 2: Fig. S4). Furthermore, mean dendritic spine head volume was similar between genotypes and this finding extended to individual spine subclasses (Fig. 8g, h). These findings suggest that ROCK2 deficiency in excitatory neurons of the mPFC and BLA does not alter dendritic spine density or morphology in these cell populations.

## Discussion

Twenty-nine protein kinase inhibitors have been used to treat human diseases, and out of these, two are pan-ROCK inhibitors: Fasudil and Ripasudil [50]. In this study, we generated a new ROCK2^fl/fl^ mouse model, and examined the impact of forebrain excitatory neuron-specific ROCK2 deficiency on cognitive function and dendritic spine morphology in the hippocampus, mPFC, and BLA. While performance on the open field and passive avoidance tests was normal, Cre/ROCK2^fl/fl^ mice displayed anxiety-like behavior in the EPM and light–dark box test. These cognitive phenotypes were associated with a number of alterations in dendritic spine density and morphology among CA1 pyramidal neurons of the hippocampus, while the dendritic spines in the mPFC and BLA appeared relatively unaffected by ROCK2 deficiency.

Previous studies demonstrated that mice treated with the pan-ROCK inhibitor Y-27632 or Fasudil spent less time in the open arms of the EPM compared to vehicle controls [32, 51]. Furthermore, neither Fasudil nor Y-27632 had significant effects on exploratory behavior or locomotor activity. These findings matched our results in a new model of ROCK2 deficiency. Saitoh et al. described no significant effect of Y-27632 on spontaneous alternations in the Y-maze, which was similar to results in ROCK2 heterozygous mice [32, 51]. Our findings support this work by showing that Cre/ROCK2^fl/fl^ mice are comparable to littermates in the passive avoidance test. Both the spontaneous alternations version of the Y-maze and the passive avoidance test can assess prelimbic prefrontal cortex-based working memory in rodents [52]. This suggests that pharmacologic inhibition or genetic reduction of ROCK2 does not negatively alter memory-type behavior in mice. Other studies revealed that mice exposed to Y-27632 did not display anxiety in the EPM but did exhibit enhanced spatial discrimination in Y-maze [53]. These contrasting results could be due to off-target or peripheral effects of Y-27632. Based on the analogous EPM phenotypes under pharmacologic pan-ROCK inhibition, ROCK2 heterozygosity, or conditional deletion of ROCK2 herein, we hypothesize that disruption of ROCK2’s function in forebrain excitatory neurons drives anxiety-like behavior in mice.

The majority of excitatory synapses in the brain exist on dendritic spines [54]. Spine shape is intricately associated with the strength of synapse transmission and the active life of a synapse [54–60]. Spines can be segregated based on their architecture as mushroom, stubby, or thin [61–64]. Mushroom spines typically exhibit a lasting voluminous head but lean neck, whereas thin spines are more active and do not feature the large head of a mushroom spine. It is hypothesized that stubby spines represent intermediary dendritic protrusions with the potential to form mushroom spines. From the three brain regions that were analyzed in this study, ROCK2 deficiency among CA1 pyramidal neurons of the hippocampus exhibited the most robust changes in dendritic spine density and morphology. Cre/ROCK2^fl/fl^ mice displayed increased density of thin spines on basal dendrites, reduced mushroom spine length and head volume on basal dendrites, and reduced mean spine head volume across all spine types on apical dendrites. Notably, an increase in thin spine density was also observed following treatment of primary rat hippocampal neurons with the pan-ROCK inhibitor Y-27632 [65]. Spine length strongly affects molecular diffusion, with the spine neck as the predominant mediator of compartmentalization, facilitating regulation of biochemical and electrical components [66]. Dendritic spine head volume is correlated with the size of the postsynaptic density, and the levels of NMDA and AMPA receptors [42–45]. However, despite the reduction in spine head volume observed in Cre/ROCK2^fl/fl^ mice, protein levels of GluN1 and GluA1 were comparable in the hippocampus of Cre/ROCK2^fl/fl^ and ROCK2^fl/fl^ littermates. It is possible that the increase in basal spine density compensated for the lack of reduction in GluN1 and GluA1 levels in Cre/ROCK2^fl/fl^. Notably, we attempted to explore two potential signaling pathways that may have been disrupted under ROCK2 depletion. Several studies have indicated that pharmacologic inhibition of ROCK2 or RNAi depletion of ROCK2 leads to a reduction in phosphorylated mammalian target of rapamycin (mTOR) and induces mTOR-based autophagy pathways [16, 18, 67]. Therefore, it is possible that ROCK2 deficiency in excitatory neurons may have altered autophagic processes in these cells, leading to changes in spine morphology or synaptic dysfunction (reviewed here [68]). However, western blots of hippocampus homogenates revealed comparable levels of phosphorylated mTOR as well as the autophagy substrate p62 among Cre/ROCK2^fl/fl^ and ROCK2^fl/fl^ mice (Additional file 2: Fig. S1). These results suggest that under these experimental conditions, genetic depletion of ROCK2 does not promote mTOR-based autophagic processes in excitatory neurons. ROCK2 can mediate actin cytoskeleton rearrangement and ultimately spine density and morphology through phosphorylation of the serine/threonine LIM domain kinase isoform 1 (LIMK1) at threonine 508, and subsequent LIMK1 phosphorylation of the actin-severing protein cofilin [26, 27]. Therefore, investigated levels of phosphorylated LIMK1 (pLIMK1) in hippocampus homogenates from Cre/ROCK2^fl/fl^ mice using biochemistry but observed no reduction in pLIMK1 (Additional file 2: Fig. S1). This was not particularly surprising because LIMK1 can be phosphorylated at threonine 508 by other kinases, including the p21-activated kinases which also regulate actin dynamics [69]. We hypothesize that compensatory mechanisms are likely involved in excitatory neurons in order to control actin cytoskeleton rearrangement in the absence of ROCK2. These compensatory mechanisms could include, but are not limited to, signaling by ROCK1 or other Rho-GTPase cascades, such as Ras-related C3 botulinum toxin substrate 1 (Rac1) or Cell division control protein 42 homolog (Cdc-42) pathways [70]. Ultimately, given the spine density and morphology phenotypes among CA1 pyramidal neurons as the result of ROCK2 deficiency, we hypothesize several synaptic alterations and network changes and discuss these below.

The complexity and polarized nature of the dendritic spine architectural changes among CA1 cells under ROCK2 deficiency likely have physiological ramifications at the cell and network level. Notably, the process of iontophoretic microinjection of Lucifer yellow is blind; therefore, we could not distinguish between deep and superficial layer CA1 pyramidal cells. Basal dendrites on CA1 pyramidal neurons receive feedforward excitation from hippocampal area CA2 as well as CA3 Schaffer collaterals, therefore changes in synaptic strength due to reduced length and head volume of basal mushroom spines could alter these cellular connections [71]. Additionally, changes in CA1 basal dendritic spine morphology could affect excitatory–inhibitory microcircuits, including inhibition by parvalbumin-expressing basket cells [72]. A gross overall reduction in dendritic spine head volume among apical dCA1 pyramidal dendrites could affect feedforward excitation from the medial and lateral entorhinal cortex as well as CA3 Schaffer collateral excitation [73]. Finally, ROCK2 deficiency could be altering electrophysiological processes and network connectivity of CA1 pyramidal neurons that are beyond the noted dendritic spine phenotypes. Both deep and superficial layer CA1 pyramidal cells provide output to several cortical areas, including the mPFC and amygdala, but also the entorhinal cortex, retrosplenial cortex, subicular complex, nucleus accumbens, lateral septum, and lateral hypothalamus [72–74]. Disruption of these network connections may have contributed to the anxiety-like behavior exhibited by Cre/ROCK2^fl/fl^ mice.

Despite the lack of dendritic spine density or morphology phenotypes in the mPFC or BLA of Cre/ROCK2^fl/fl^ mice, it is possible that ROCK2 deficiency among excitatory neurons in these brain regions rendered the cells dysfunctional. However, it is also possible that projections from the vCA1 to the lateral hypothalamic area (LHA), but not to the BLA, is modulating the anxiety-like behavior in ROCK2 deficient mice. Elegant optogenetic studies by Jimenez et al*.* demonstrated that activation of vCA1 axon terminals in LHA increased anxiety and avoidance behaviors, showing that the vCA1-LHA pathway is a direct route by which the hippocampus can regulate anxiety [75]. From this standpoint, the robust increases in spine length among vCA1 pyramidal cells in Cre/ROCK2^fl/fl^ mice could alter the activity of these cells’ projections to the LHA, thus influencing the reactions in anxiogenic environments.

Protein prenylation adds lipid molecules, or isoprenoids, to proteins, and this critical post-translational modification controls a variety of cellular signaling pathways [76]. Over a hundred proteins undergo protein prenylation, including a number of GTPases. Prenylation anchors small GTPases, including RhoA, to membranes which is required for most GTPase signaling functions [77]. Two enzymes, farnesyltransferase (FT) and geranylgeranyltransferase type I (GGT), are required for the prenylation process, and the significance of these enzymatic processes is highlighted by elegant studies in brain showing that genetic reduction of FT or GGT alters hippocampal plasticity, dendritic spine density, learning and memory behaviors, and reaction to anxiogenic environments [78–82]. GTP-bound RhoA can activate ROCK1 or ROCK2, therefore it is possible that in the absence of ROCK2, RhoA-induced activity of ROCK1 is altered in excitatory neurons, accounting in part for the observed dendritic spine phenotypes.

## Conclusions

Previously, we demonstrated that ROCK2^+/−^ mice exhibited alterations in dendritic spine morphology among layers 2/3 pyramidal neurons in the prelimbic mPFC, including increased apical spine length and spine head diameter [31]. However, in this report no significant changes in spine density or morphology were observed among these cells in Cre/ROCK2^fl/fl^ mice compared to ROCK2^fl/fl^ littermates. These contradictory findings may be explained by the difference in genetic models used to assess the role of ROCK2 on spine structure. Cre/ROCK2^fl/fl^ mice lack ROCK2 exclusively in excitatory neurons in the mPFC, whereas all cell types in the brain were heterozygous for ROCK2 in ROCK2^+/−^ mice. Henceforth, other cells, like interneurons, that could affect spine dynamics on excitatory neurons exhibited reduction of ROCK2 in ROCK2^+/−^ mice. Moreover, ROCK2^+/−^ mice developed from an embryonic state under ROCK2 heterozygosity, whereas Cre/ROCK2^fl/fl^ mice lost ROCK2 from excitatory neurons approximately 21 days post-birth [35]. A primary goal of this study was to provide an initial description of the ROCK2^fl/fl^ mice and illustrate the utility of this new model system to investigate ROCK2 functions in a cell-type specific manner. The ROCK2^fl/fl^ mice will offer an innovative approach for future studies to unravel the physiological and pathological roles of ROCK2 in brain as well as other organs.

## Supplementary Information

**Additional file 1: Table S1.** The number of neurons, dendrites, dendritic length, and spines analyzed per animal in each brain region.

**Additional file 2: Figure S1.** Biochemical analysis of hippocampus homogenates from Cre/ROCK2^fl/fl^ mice. **Figure S2.** Medial prefrontal cortex spine length and head diameter in Cre/ROCK2^fl/fl^ mice. **Figure S3.** Medial prefrontal cortex spine volume in Cre/ROCK2^fl/fl^ mice. **Figure S4.** Basolateral Amygdala spine length and head diameter in Cre/ROCK2^fl/fl^ mice.

## Data Availability

All data generated or analyzed during this study are included in the published article and its additional information files.

## Abbreviations

AMPA: α-Amino-3-hydroxy-5-methyl-4-isoxazolepropionic acid
BLA: Basolateral amygdala
DAPI: 4ʹ,6-Diamidino-2-phenylindole
EPM: Elevated plus maze
GAPDH: Glyceraldehyde-3-phosphate dehydrogenase
mPFC: Medial prefrontal cortex
NMDA: *N*-Methyl-d-aspartate
PBS: Phosphate-buffered saline
PFA: Paraformaldehyde
ROCK2: Rho-associated kinase isoform 2
