# Supplementary material for: Conditional deletion of ROCK2 induces anxiety-like behaviors and alters dendritic spine density and morphology on CA1 pyramidal neurons
Source: Mol Brain. 2021 Nov 18;14:169. doi: 10.1186/s13041-021-00878-4 (PMC8600782; doi:10.1186/s13041-021-00878-4)
Supplement: Supplementary file 1 — Additional file 1: Table S1. The number of neurons, dendrites, dendritic length, and spines analyzed per animal in each brain region. [file 13041_2021_878_MOESM1_ESM.docx]

|  | **# neurons** | **# dendrites (a, b)** | **dendritic length (a, b)** | **# spines (a, b)** |
| --- | --- | --- | --- | --- |
| **dCA1** |  |  |  |  |
| R2^fl/fl^ | 10 | 10 (4, 6) | 366.8 (148.6, 218.2) | 1144 (495, 649) |
| R2^fl/fl^ | 5 | 10 (5, 5) | 399.6 (199.0, 200.6) | 1322 (694,628) |
| R2^fl/fl^ | 6 | 11 (5, 6) | 437.1 (203.6, 233.5) | 1593 (769, 824) |
| R2^fl/fl^ | 7 | 10 (5, 5) | 350.5 (182.1, 168.4) | 1130 (593, 537) |
| R2^fl/fl^ | 7 | 10 (5, 5) | 382.6 (187.4, 195.2) | 1266 (628, 638) |
| Cre/R2^fl/fl^ | 9 | 12 (5,7) | 387.5 (184.2, 203.3) | 1392 (591, 801) |
| Cre/R2^fl/fl^ | 7 | 11 (5, 6) | 399.4 (170.4, 229.0) | 1377 (559, 818) |
| Cre/R2^fl/fl^ | 9 | 11 (5, 6) | 321.0 (155.5, 165.5) | 1103 (520, 583) |
| Cre/R2^fl/fl^ | 10 | 12 (6, 6) | 426.0 (202.4, 223.6) | 1364 (633, 731) |
| Cre/R2^fl/fl^ | 9 | 11 (5,6) | 399.7 (192.1, 207.6) | 1662 (768,894) |
| Cre/R2^fl/fl^ | 7 | 12 (6,6) | 446.1 (224.9, 221.2) | 1831 (978, 853) |
| Cre/R2^fl/fl^ | 5 | 10 (5,5) | 324.2 (159.8, 164.6) | 1429 (723,706) |
| Cre/R2^fl/fl^ | 8 | 11(6,5) | 365.9 (206.4, 159.5) | 1399 (795,604) |
|  | **99** | **141 (67, 74)** | **5006 (2416, 2590)** | **18012 (8746, 9266)** |
| **vCA1** |  |  |  |  |
| R2^fl/fl^ | 7 | 10 (5,5) | 370.1 (189.5, 180.6) | 1407 (737, 670) |
| R2^fl/fl^ | 5 | 10(5,5) | 354.5 (172.0, 182.5) | 1369 (680, 689) |
| R2^fl/fl^ | 5 | 10(5,5) | 335.1 (168.5, 166.6) | 1301 (685, 616) |
| R2^fl/fl^ | 5 | 9 (5, 4) | 350.7 (199.5, 151.2) | 1050 (615, 435) |
| R2^fl/fl^ | 8 | 12 (6,6) | 406.5 (203.3, 203.2) | 1492 (788, 704) |
| Cre/R2^fl/fl^ | 5 | 10 (5,5) | 311.5 (172.8, 138.7) | 1182 (760, 422) |
| Cre/R2^fl/fl^ | 7 | 10 (5, 5) | 359.5 (195.6, 163.9) | 1378 (728, 650) |
| Cre/R2^fl/fl^ | 5 | 10 (5,5) | 334.0 (165.9, 168.1) | 1250 (545, 705) |
| Cre/R2^fl/fl^ | 8 | 10 (6, 4) | 338.8 (213.0, 125.8) | 1399 (908, 491) |
| Cre/R2^fl/fl^ | 5 | 10 (5,5) | 350.1 (176.5, 173.6) | 1446 (664, 782) |
| Cre/R2^fl/fl^ | 4 | 7 (3,4) | 264.9 (122.4, 142.5) | 951 (439, 512) |
|  | **64** | **108 (55, 53)** | **3776 (1979, 1797)** | **14225 (7549, 6676)** |
| **mPFC** |  |  |  |  |
| R2^fl/fl^ | 6 | 9 (4, 5) | 368.6 (179.1, 189.5) | 1052 (542, 510) |
| R2^fl/fl^ | 7 | 11 (5, 6) | 527.9 (242.2, 285.7) | 1185 (533, 652) |
| R2^fl/fl^ | 6 | 9 (4, 5) | 423.5 (179.8, 243.7) | 1007(475, 532) |
| R2^fl/fl^ | 4 | 8 (4, 4) | 331.4 (161.7, 169.7) | 1028 (520, 508) |
| R2^fl/fl^ | 7 | 11 (5, 6) | 419.8 (193.6, 226.2) | 1234 (574, 660) |
| Cre/R2^fl/fl^ | 8 | 13 (5,8) | 544.1 (196.7, 347.4) | 1382 (503, 879) |
| Cre/R2^fl/fl^ | 6 | 8 (3, 5) | 340.5 (122.3, 218.2) | 799 (251, 548) |
| Cre/R2^fl/fl^ | 6 | 10 (5, 5) | 458.7 (230.1, 228.6) | 1133 (590, 543) |
| Cre/R2^fl/fl^ | 9 | 11 (5, 6) | 528.0 (224.5, 303.5) | 1616 (638, 978) |
| Cre/R2^fl/fl^ | 6 | 10 (5, 5) | 400.7 (213.6, 187.1) | 1083 (572, 511) |
| Cre/R2^fl/fl^ | 9 | 11 (5, 6) | 463.3 (213.2, 250.1) | 1313 (678, 635) |
| Cre/R2^fl/fl^ | 6 | 12 (5, 7) | 441.0 (211.6, 229.4) | 1104 (433, 671) |
|  | **80** | **123 (55, 68)** | **5248 (2368, 2879)** | **13936 (6309, 7627)** |
| **BLA** |  |  |  |  |
| R2^fl/fl^ | 4 | 4 | 138.8 | 366 |
| R2^fl/fl^ | 10 | 14 | 485.5 | 1404 |
| R2^fl/fl^ | 11 | 14 | 515.5 | 1499 |
| R2^fl/fl^ | 6 | 6 | 159.9 | 440 |
| Cre/R2^fl/fl^ | 9 | 10 | 337.6 | 1008 |
| Cre/R2^fl/fl^ | 8 | 10 | 371.7 | 974 |
| Cre/R2^fl/fl^ | 6 | 9 | 331.6 | 1238 |
| Cre/R2^fl/fl^ | 8 | 9 | 343.7 | 909 |
| Cre/R2^fl/fl^ | 4 | 5 | 162.9 | 772 |
| Cre/R2^fl/fl^ | 10 | 11 | 413.7 | 1105 |
|  | **76** | **92** | **3261** | **9715** |

**Table S1**. Dendritic length is in µm. For BLA dendrites, values are a combination of apical and basal dendrites. Total values per group are bolded. a = apical; b = basal; dCA1, dorsal CA1; vCA1, ventral CA1; mPFC, medial prefrontal cortex; BLA, basolateral amygdala. R2^fl/fl^, ROCK2^fl/fl^; Cre/R2^fl/fl^, Cre/ROCK2^fl/fl^.
