# Supplementary material for: Conditional deletion of ROCK2 induces anxiety-like behaviors and alters dendritic spine density and morphology on CA1 pyramidal neurons
Source: Mol Brain. 2021 Nov 18;14:169. doi: 10.1186/s13041-021-00878-4 (PMC8600782; doi:10.1186/s13041-021-00878-4)
Supplement: Supplementary file 2 — Additional file 2: Figure S1. Biochemical analysis of hippocampus homogenates from Cre/ROCK2fl/fl mice. Figure S2. Medial prefrontal cortex spine length and head diameter in Cre/ROCK2fl/fl mice. Figure S3. Medial prefrontal cortex spine volume in Cre/ROCK2fl/fl mice. Figure S4. Basolateral Amygdala spine length and head diameter in Cre/ROCK2fl/fl mice. [file 13041_2021_878_MOESM2_ESM.docx]

**Additional file 2**

**
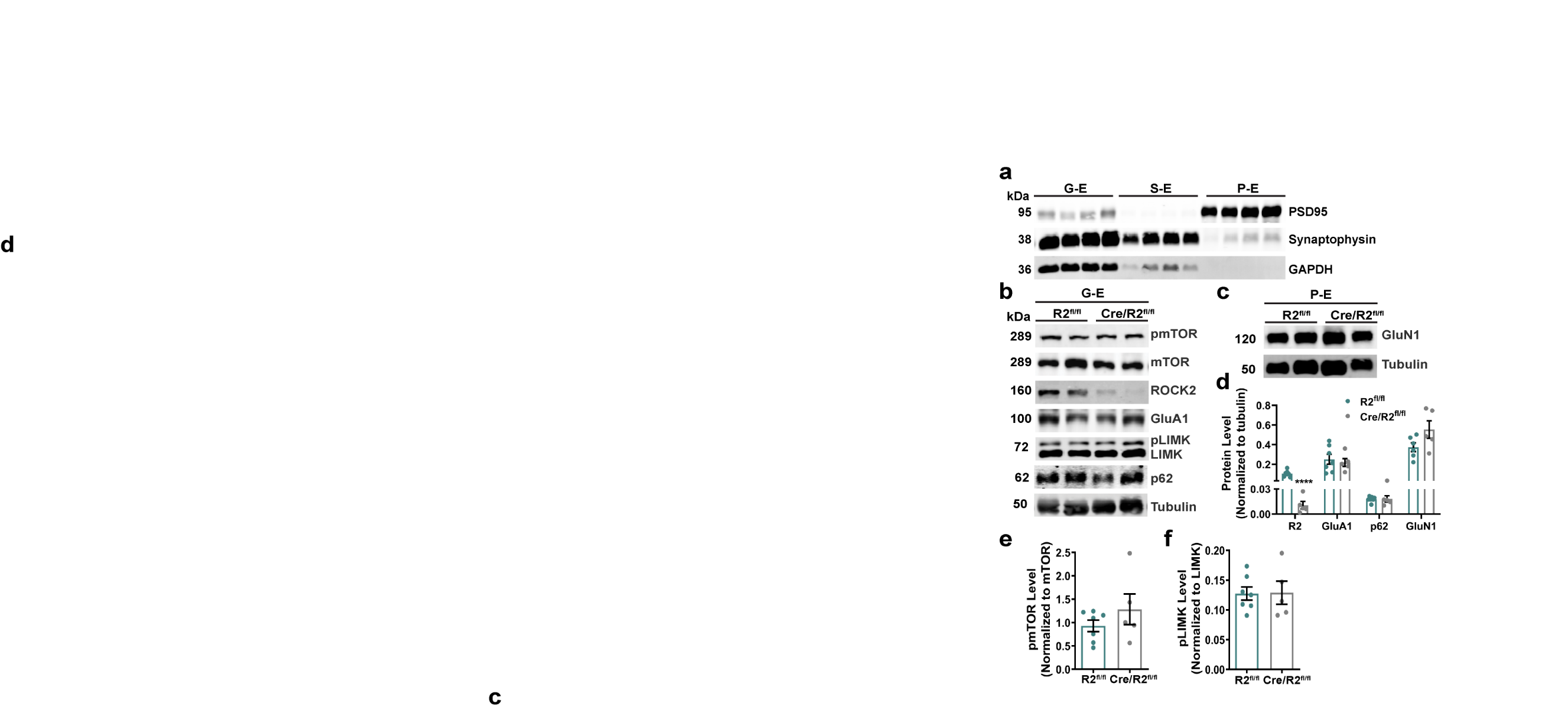
**

**Figure S1. Biochemical analysis of hippocampus homogenates from Cre/ROCK2^fl/fl^ mice. (a)**Representative western blot of GAPDH-enriched (G-E), synaptophysin-enriched (S-E), and PSD95-enriched (P-E) fractions from ROCK2^fl/fl^ mouse hippocampus for PSD95, synaptophysin, and GAPDH. **(b-c)** Representative western blots from G-E **(b)** and P-E fractions **(c)** of ROCK2^fl/fl^ and Cre/ROCK2^fl/fl^ mice. **(d)** Densitometry analysis of protein levels from G-E and P-E fractions of ROCK2^fl/fl^ and Cre/ROCK2^fl/fl^. Protein levels were normalized to tubulin. Cre/ROCK2^fl/fl^ G-E fraction showed decreased ROCK2 levels in the hippocampus (t(10)= 6.283, ****p<0.0001). There were no significant differences in GluA1 (t(10)= 0.4755, p=0.6446), p62 (t(10)= 0.1041, p=0.9191), or GluN1 (t(9)= 1.920, p=0.0871) protein levels between ROCK2^fl/fl^ and Cre/ROCK2^fl/fl^ mice. **(e)** pmTOR protein levels, normalized to mTOR, were similar in Cre/ROCK2^fl/fl^ compared to ROCK2^fl/fl^ mice (t(10)= 1.143, p=0.2795). **(f)** pLIMK levels were normalized to LIMK protein levels. pLIMK levels were comparable between ROCK2^fl/fl^ and Cre/ROCK2^fl/fl^ mice (t(10)= 0.6620, p=0.9485). N= 7 ROCK2^fl/fl^ mice (3 M, 4 F) and 5 Cre/ROCK2^fl/fl^ (3 M, 2 F) mice at 8-9 months. Unpaired t-tests were used for all comparisons. ROUT outlier test identified one outlier in NMDAR1 data set (ROCK2^fl/fl^: 1.509259). Each point represents one mouse and the error bars indicate the standard error of the mean. R2, ROCK2; R2^fl/fl^, ROCK2^fl/fl^; Cre/R2^fl/fl^, Cre/ROCK2^fl/fl^.


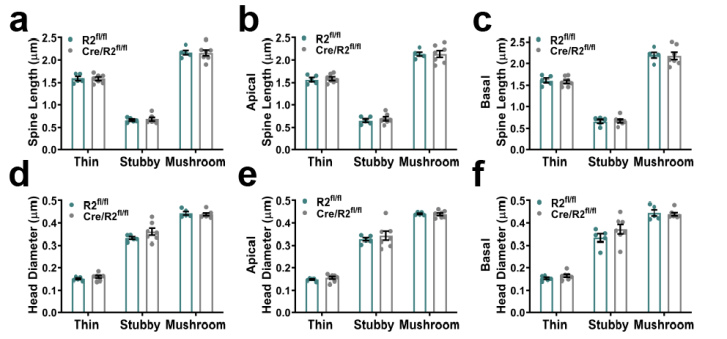


**Figure S2. Medial prefrontal cortex spine length and head diameter in Cre/ROCK2^fl/fl^ mice. (a-c)** There were no differences in **(a)** combined dendritic mean, **(b)** apical, and **(c)** basal spine lengths of thin, stubby, or mushroom spines between groups. **(d-f)** There were no differences in head diameter for **(d)** all dendrites combined, as well as for **(e)** apical and **(f)** basal spines between groups. N= 5 ROCK2^fl/fl^ mice (2 M, 3 F) and 7 Cre/ROCK2^fl/fl^ (4 M, 3 F) mice at 8-9 months. Unpaired t-tests were used for all comparisons. Each point represents one mouse and the error bars indicate the standard error of the mean. R2^fl/fl^, ROCK2^fl/fl^; Cre/R2^fl/fl^, Cre/ROCK2^fl/fl^.


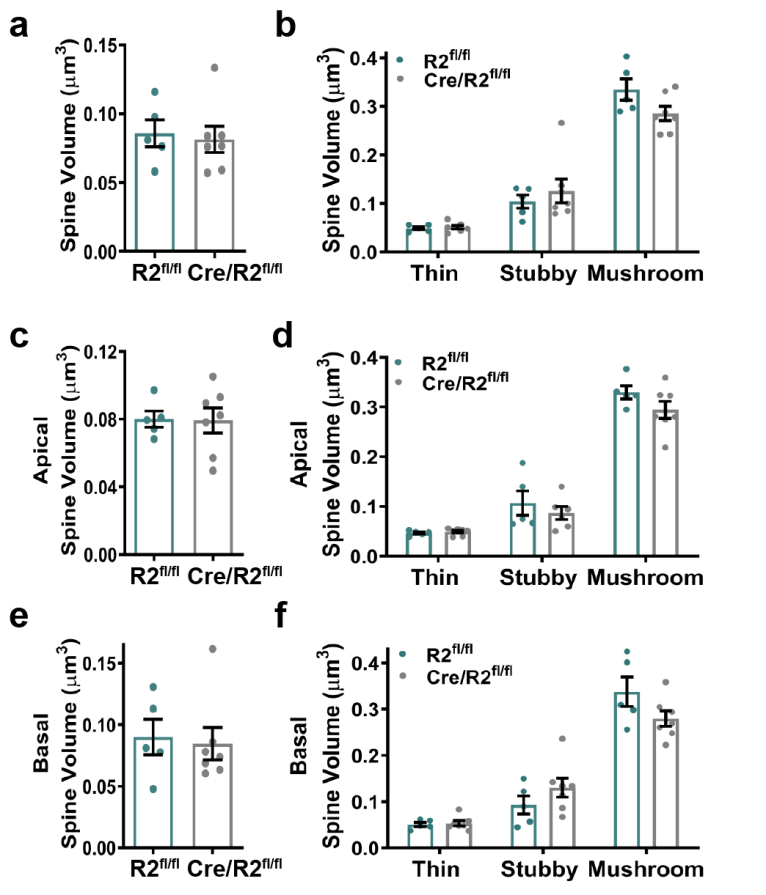


**Figure S3. Medial prefrontal cortex spine volume in Cre/ROCK2^fl/fl^ mice.** Cre/ROCK2^fl/fl^ mice had similar **(a)** overall spine volume and **(b)** spine volume of thin, stubby, or mushroom subclasses compared to ROCK2^fl/fl^ mice. There were no differences in **(c)** apical spine volume and **(d)** volume of apical spine subclasses between groups. Basal **(e)** spine volume and **(f)** volume of basal spine subclasses were similar between groups. N= 5 ROCK2^fl/fl^ mice (2 M, 3 F) and 7 Cre/ROCK2^fl/fl^ (4 M, 3 F) mice at 8-9 months. Unpaired t-tests were used for all comparisons. Each point represents one mouse and the error bars indicate the standard error of the mean. R2^fl/fl^, ROCK2^fl/fl^; Cre/R2^fl/fl^, Cre/ROCK2^fl/fl^.


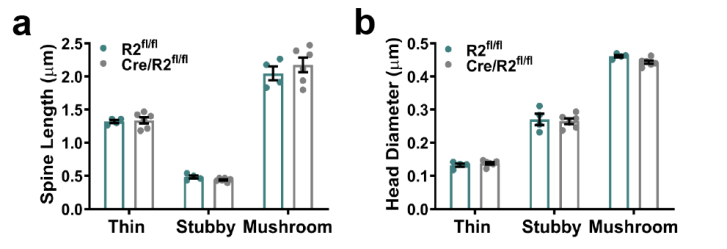


**Figure S4. Basolateral Amygdala spine length and head diameter in Cre/ROCK2^fl/fl^ mice.** There were no differences in **(a)** spine length or **(b)** head diameter of thin, stubby, or mushroom spines between groups. N= 4 ROCK2^fl/fl^ mice (1 M, 3 F) and 6 Cre/ROCK2^fl/fl^ (4 M, 2 F) mice at 8-9 months. Unpaired t-tests were used for all comparisons. Each point represents one mouse and the error bars indicate the standard error of the mean. R2^fl/fl^, ROCK2^fl/fl^; Cre/R2^fl/fl^, Cre/ROCK2^fl/fl^.
